# Integrating Digital Technologies Into Biochemistry Education: A Decade of Efforts, Pandemic Impacts, and Emerging Insights

**DOI:** 10.1002/bmb.70038

**Published:** 2026-02-14

**Authors:** Francis Pereira‐Dias, Marina Bazzo de Espíndola

**Affiliations:** ^1^ Programa de Pós‐graduação Em Educação Científica e Tecnológica Federal University of Santa Catarina Florianópolis Brazil; ^2^ Departamento de Metodologia de Ensino, Programa de Pós‐Graduação Em Educação Científica e Tecnológica Federal University of Santa Catarina Florianópolis Brazil

**Keywords:** augmented reality, biochemistry education, COVID‐19, critical perspective on the use of DICTs, gamification, integration of digital information and communication technologies (DICT), platforms, virtual laboratory

## Abstract

This review critically examines the integration of Digital Information and Communication Technologies (TDICs) in biochemistry education over the past decade, highlighting both the benefits and challenges from a critical theoretical perspective. A systematic review was conducted to identify relevant literature, followed by thematic analysis and a detailed synthesis of the findings. Grounded in Feenberg's critical theory of technology and Selwyn's scholarship on education and digital technology, this review examines the implications of virtual laboratories, augmented reality, gamification, and online platforms in biochemistry education, as well as their implications related to the pandemic. We observed that digital technologies can enhance certain aspects of student engagement and learning outcomes; however, they can also hinder equitable access and hands‐on laboratory skills. This review also highlights the key elements of critical reflection on the socio‐political and ethical implications of digital technologies in biochemistry education, with a particular focus on pandemic‐era concerns, including data privacy, algorithmic bias, and the commercialization of teaching practices. Future research should focus on these dimensions to ensure that technological advancements do not perpetuate or amplify educational inequities.

## 1 Introduction

Biochemistry bridges life and chemical sciences, providing critical insights into the molecular processes that drive living organisms. Additionally, in healthcare, it facilitates the understanding and treatment of diseases, as well as the development of pharmaceuticals [1, 2]. Biochemistry improves crop yield and resilience through genetic modification and pest control [3]. Nutrition science relies on biochemistry to unravel metabolic pathways and understand the functions of nutrients, which are essential for developing dietary guidelines and interventions [4]. Environmental science benefits from biochemical approaches to understand pollutant degradation and ecosystem functions [5]. Biochemistry is a fundamental subject that helps students develop a comprehensive understanding of both biological and chemical foundations necessary to address these critical fields [6]. Incorporating biochemistry into educational curricula is crucial for preparing students to address the scientific, societal, and ethical challenges of the modern era [2, 4, 6].

However, the biochemistry subject often intimidates students due to its complex, voluminous content, foundational chemistry prerequisites, and the need for abstract thinking [7]. Concepts such as metabolic pathways, bioenergetics, and biomolecular structures are considered particularly challenging. Knight and Wood [8] emphasize that biochemistry's abstract nature and integration of multiple scientific disciplines present significant cognitive challenges for students. Similarly, Cornely [9] observes that the complexity and volume of biochemical content can overwhelm students, hindering their ability to grasp the broader context or practical relevance of their learning. Berg et al. [1] discuss the difficulties students face in developing the spatial reasoning required to understand three‐dimensional molecular structures, which is essential for mastering biochemistry.

**FIGURE 1:**
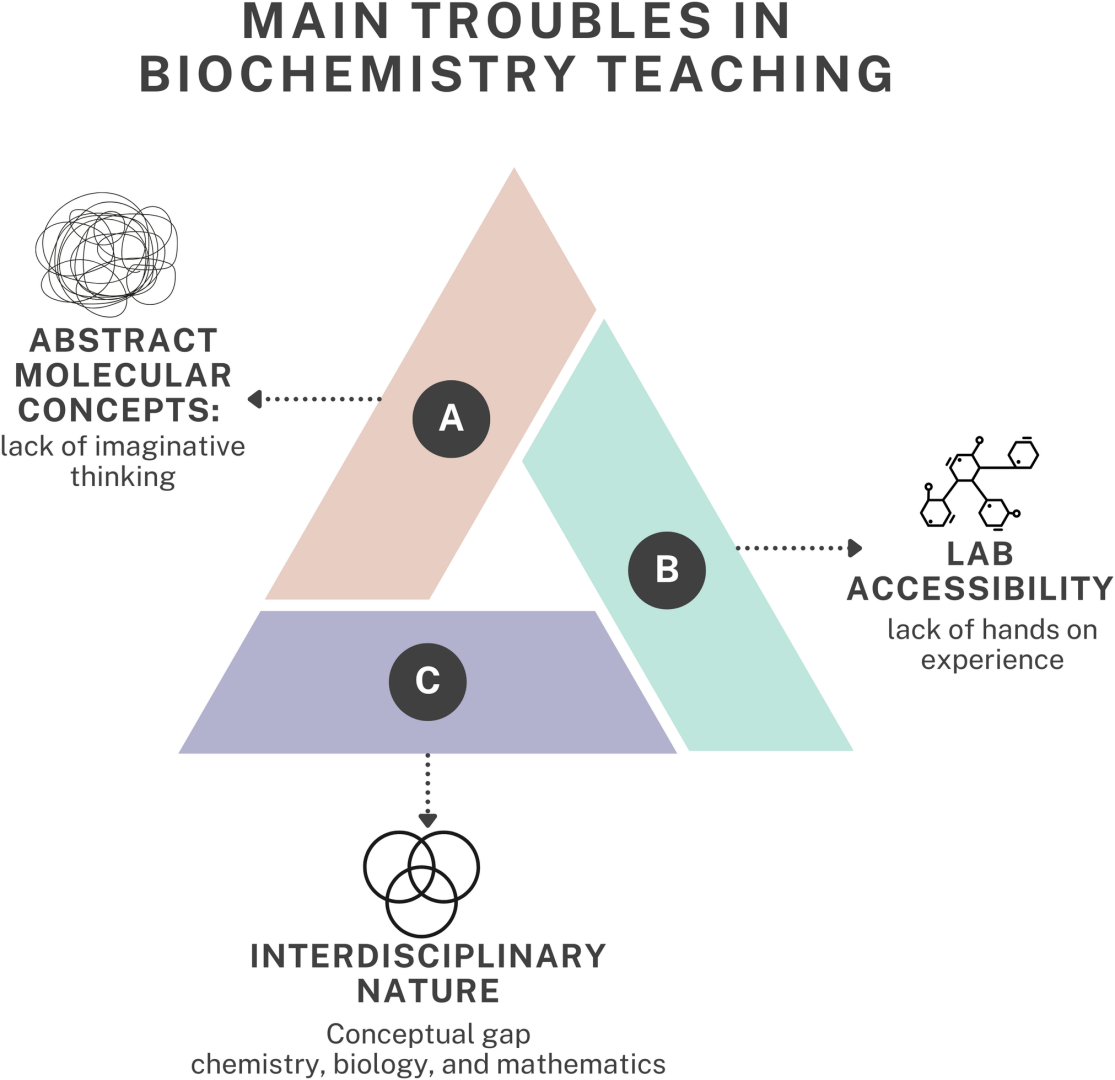
A conceptual map was developed to illustrate key challenges in biochemistry education, including its interdisciplinary scope, restricted lab access, and the abstract nature of molecular concepts.

Despite the relevance of biochemistry, educators often struggle to convey abstract concepts to students (Figure 1a) or teach 3D structures and molecular pathways, which require imaginative thinking and can be overwhelming for students (Figure 1a).

In addition, Biochemistry teaching is sometimes found to be more challenging to understand due to the lack of practical experiments and limited lab accessibility (Figure 1b); this is particularly the case in many underprivileged schools, where resource constraints restrict access to laboratory equipment (Figure 1b). Limited resources for hands‐on lab experience often leave students without practical skills essential for the field.

Also, the interdisciplinary nature of biochemistry makes it difficult for many students to fully understand (Figure 1c) and contextualize the learned knowledge to the application [10]. The convergence of chemistry, biology, and mathematics can make biochemistry seem fragmented, leading to conceptual gaps [11, 12]. Finally, as shown in Figure 1c, the interdisciplinary nature of biochemistry also creates a steep learning curve, as students must simultaneously master chemistry, biology, and mathematics [10], making biochemistry seem fragmented and leading to conceptual gaps.

Despite the increasing implementation of technology in biochemistry education, challenges remain in ensuring its integration is not merely reproductive or mechanistic. A critical issue lies in addressing the disparities in access to digital tools, which can exacerbate existing inequalities among students. Over the past few decades, the integration of technology in education has become increasingly crucial for fostering citizenship, particularly from the perspectives of Critical Pedagogy and the Critical Theory of Technology. These frameworks emphasize the importance of using technology not as a passive tool but as a means to transform learning environments, promote critical thinking, and prepare students for active social participation. In biochemistry education, educators must intentionally question and evaluate the technologies they incorporate, ensuring they foster a more profound understanding, encourage inquiry, and bridge theoretical knowledge with practical skills. By doing so, technology becomes a transformative force that addresses conceptual gaps and aligns with the ethical and social responsibilities of modern education.

Amidst these challenges, many researchers have explored methodologies to mitigate the difficulties in teaching biochemistry. We asked ourselves if technology addresses the main troubles in biochemistry teaching (Figure 1). In this review, we analyze the application of technology in biochemistry education, focusing on the how, when, and why of its use. We examine if there are works that incorporate Critical Pedagogy and Technology Integration assessment [13, 14], emphasizing the need for digital tools to improve pedagogy, create inclusive learning environments, and prepare biochemists for the complexities of the 21st century. Responsible integration of technology in biochemistry education can enhance student engagement, bridge theory with practical application, and promote collaborative problem‐solving while respecting ethical principles.

## 2 Methodology: Review of Literature and Data Synthesis

### 2.1 Literature Search Strategy

We employed a comprehensive search strategy using electronic databases such as Web of Science, Scopus, and ERIC, guided by the structured approach outlined by Bramer et al. [15]. The search terms “Biochemistry Education” and “Technology,” among others, were used with Boolean operators to ensure the retrieval of relevant literature on the impact of digital technology on biochemistry education.

### 2.2 Selection Criteria

Our selection criteria prioritized peer‐reviewed articles published, including time (2015–2024) and the intersection of biochemistry education and digital technology use. Articles during the pandemic were accepted. Book reviews, short communications, and articles outside of the languages (Portuguese, English, and Spanish) were rejected. Articles that did not discuss Biochemistry Education as a Subject of interest or did not report the use of technology were also dismissed. This selection strategy aligns with Moher et al. [16] and Romanowski [17], who underscore the importance of including high‐quality, relevant studies for systematic reviews. A total of 60 articles were found and thoroughly examined. No ethical approval was required as this study is theoretical and did not involve human participants or animals.

### 2.3 Data Extraction and Synthesis

Following Booth [18], the data extraction process systematically retrieved information like authors, publication year, study design, digital technologies employed, pedagogical approaches, and critical findings. A thematic analysis, guided by Bardin [19], was conducted to identify overarching themes and gaps in the literature, revealing how digital technology impacted biochemistry education during the pandemic, as shown in Table 1. The synthesis process adhered to Minayo's [78] principles, integrating findings cohesively and critically. The data analysis encompassed both qualitative and quantitative aspects. When possible, statistical analysis was performed using the Pearson correlation coefficient. The Pearson correlation measures the linear relationship between two variables, ranging from −1 (perfect negative relationship) to 1 (perfect positive relationship), with 0 indicating no linear relationship at all. This method assessed the strength and direction of associations between variables, providing a deeper understanding of their interdependencies [79].

**TABLE 1:** An Overview of Key Studies in Biochemistry Education summarizes the references, biochemistry subjects, study focuses, and key findings of various studies that explore the use of online platforms in biochemistry education.

Code	References	Biochemistry subject	Study focus and Key findings
S1	Ahern [20]	General biochemical principles, metabolic pathways, and molecular biology	Presents a comprehensive overview of the online biochemistry courses at Oregon State University. The strengths lie in the creative and accessible teaching methods; however, there are noted limitations in interactivity, student engagement with digital technologies, and performance assessment.
S2	Silva de Alcantara et al. [21]	Functional and structural characteristics of carbohydrates, lipids, and proteins.	Describe the development and evaluation of BioDomínio, a biochemistry board game that effectively enhances student engagement and understanding through a fun and interactive approach.
S3	Almendro Vedia et al. [22]	Experiment for lipid extraction	Describe a method for producing giant unilamellar vesicles (GUVs) using lipids extracted from chicken eggs and a smartphone, making advanced biochemical experiments accessible in general educational settings.
S4	Argüello and Dempski [23]	Protein visualization	A novel AR‐based approach enables undergraduates to visualize protein structures, enhancing engagement and comprehension in biochemistry education.
S5	Barrow et al. [24]	Citric acid cycle	Demonstrates the effectiveness of VR in enhancing engagement and understanding of the citric acid cycle among undergraduate biochemistry students.
S6	Bennie et al. [25]	Enzyme catalysis	Explores the use of iMD‐VR to teach enzyme catalysis, highlighting improved engagement and understanding among students.
S7	Berry and Board [26]	Protein structure visualization	Describes the use of augmented reality to create interactive 3D visualizations of protein structures, enhancing student engagement and understanding in biochemistry education.
S8	Bibic et al. [27]	Biochemistry of venoms	Development and implementation of Bug Off Pain, a VR game designed to educate the public and high school students about the biochemistry of spider venoms and chronic pain. The game has received positive feedback for its engaging and educational content, demonstrating significant improvements in learning outcomes.
S9	Shu et al. [28]	Hepatic biotransformation	Implementation of Rain Classroom, a mobile learning tool integrated with WeChat, to enhance biochemistry teaching. The study showed improved learning outcomes and student engagement compared with traditional methods.
S10	Booth et al. [29]	Metabolism (Purine Biosynthesis, Regulation of Cellular Respiration, Glycolysis, Tricarboxylic Acid Cycle, Electron Transport Chain, and Fermentation)	Demonstrates that incorporating online computational systems and dynamical models into upper‐division undergraduate biochemistry courses significantly enhances students' understanding of metabolism, thereby improving their cognitive and technical skills.
S11	Bussey and Orgill [11]	Protein Translation (Shine‐Dalgarno Sequence mRNA)	This paper examines how biochemistry students interpret and learn from dynamic animations of protein translation, with a primary focus on the Shine‐Dalgarno sequence. It highlights the influence of animation design on student attention and understanding.
S12	Castro de Jesus and Cabral [30]	Biochemistry and molecular biology to design a synthetic biology product	Using synthetic biology challenges to teach molecular biology online during the COVID‐19 pandemic, showing positive outcomes in student engagement, learning, and satisfaction.
S13	Chou et al. [31]	Biochemistry and Drug Development	Summarizes a conference session that presented innovative tools and methods for teaching biochemistry and molecular biology, including undergraduate research experiences, music, and multimedia technologies, showing their potential to enhance student engagement and learning outcomes.
S14	Coan et al. [32]	Protein and DNA visualization	Explores the use of virtual reality in teaching biochemistry, demonstrating high student engagement and effectiveness in molecular visualization, though technical barriers remain.
S15	Constible [33]	Biochemistry laboratory practices, including enzyme kinetics, protein quantification, and fundamental mathematical skills	Demonstrates the effective use of online simulations to teach biochemistry laboratory content during the COVID‐19 pandemic, ensuring continuity of education and maintaining high student engagement and performance levels.
S16	Daina et al. [34]	Biochemistry and Drug Development	The Drug Design Workshop is an educational web‐based tool designed to introduce high school students and the general public to computer‐aided drug design. It provides hands‐on experience with professional bioinformatics tools through an accessible and engaging platform.
S17	Dash [35]	Biochemistry for Medical	Evaluates the use of Google Classroom as an LMS in teaching biochemistry to first‐year medical students, showing positive outcomes in student engagement and accessibility, especially in resource‐limited settings.
S18	ElGolli‐Bennour et al. [36]	Biochemistry laboratory for dental medicine	Evaluates dental medicine students' perceptions of remote biochemistry laboratories during the COVID‐19 pandemic, highlighting a preference for hybrid learning and identifying challenges such as internet connectivity issues
S19	Ferreira et al. [37]	Biochemistry, metabolism, and glycolysis	Investigates the role of online communication tools, particularly discussion forums, in the teaching and learning processes of an online biochemistry course, emphasizing the importance of interaction and collaboration among students and instructors for effective learning.
S20	Gan et al. [38]	Redox reactions	Development and implementation of an augmented reality tool to visualize and interact with the generation of oxygen gas from hydrogen peroxide and bleach, demonstrating its effectiveness in enhancing student learning and confidence in chemical handling.
S21	Garcia‐Bonete et al. [39]	Symmetry and biomolecular structure–function (Bacterioferritin)	Understanding the concept of TPAK, the article provides a practical guide for educators to develop VR and AR exercises for teaching structural biology, demonstrating the feasibility of creating immersive educational tools with accessible software and highlighting the positive impact on student engagement and learning.
S22	Vega Garzón et al. [40]	Metabolic pathways	Describes the development and implementation of the VRMET application for teaching metabolic pathways using virtual reality, highlighting its effectiveness in enhancing visual literacy and understanding of biochemical processes among university students.
S23	Govindarajan and Rajaragupathy [41]	Biochemistry for Medical	Evaluate the implementation and effectiveness of online team‐based learning in teaching biochemistry to first‐year MBBS students during the COVID‐19 pandemic, highlighting improvements in student engagement, knowledge, and team skills despite technical and logistical challenges.
S24	Guzmán and Joseph [42]	Anaerobic Digestion	Presents the development and application of a web‐based virtual lab for teaching anaerobic digestion processes, demonstrating its effectiveness in enhancing practical learning experiences and accessibility for engineering and science students.
S25	Haile [43]	Biochemistry of food	Discusses the implementation of blogs in a flipped classroom model for a first‐year seminar course on food chemistry, highlighting the enhancement of student engagement and the creation of an interactive online learning community through public blogs.
S26	Higgins et al. [44]	General Biochemistry	Evaluate the use of PeerWise to embed retrieval practice in an undergraduate biochemistry course, showing positive correlations between student engagement with the tool and improved performance. However, some students encountered challenges with the quality of questions and a lack of moderation.
S27	Hoog et al. [45]	Structural biology, focusing on molecular visualization of biomolecules like GFP.	Discusses the rapid deployment of a smartphone‐based AR tool for teaching structural biology, demonstrating its effectiveness in enhancing student engagement and understanding of molecular structures in a remote learning environment, particularly during the COVID‐19 pandemic.
S28	Howell et al. [46]	Nucleic acids	Demonstrates the effectiveness of 3D printed models and interactive modules in improving undergraduate biochemistry students' understanding of DNA structure–function relationships.
S29	Howell et al. [47]	Protein structure visualization and function	Showcases the application of 3D printed models in undergraduate biochemistry education. These interactive modules significantly enhance the understanding and retention of protein structure and function concepts by addressing specific student misconceptions.
S30	Jeyarajaguru [48]	Biochemistry Laboratory (*laboratory calculations and prepare various solutions, qualitative and quantitative analysis of biomolecules like carbohydrates, amino acids, and lipids, determine the saponification value of oils, among others*)	Development and implementation of a virtual curation lab using Google Sites for a Principles of Biochemistry course, demonstrating its effectiveness in enhancing student engagement and learning during the COVID‐19 pandemic.
S31	Souza Junior et al. [49]	Biochemistry topics include amino acids, peptides, proteins, hemoglobin, enzymes, enzyme kinetics and inhibition, and vitamins.	Application of blended learning with gamification in a Flipped Classroom model to enhance the teaching of biochemistry, resulting in increased student interest and improved
S32	Kamber [50]	General Biochemistry	Effectiveness of virtual oral examinations in an undergraduate biochemistry course, demonstrating increased student engagement and understanding through personalized feedback during the COVID‐19 pandemic.
S33	Kinsella et al. [51]	General Biochemistry	Demonstrates that pre‐lecture screencasts combined with MCQs can enhance student engagement and improve academic performance in large biochemistry classes.
S34	Kubbara et al. [52]	Biochemistry for Medical	This study examined the effectiveness of online case‐based learning for teaching biochemistry to medical students at King Abdulaziz University during the COVID‐19 pandemic. It demonstrated significant improvements in student performance and engagement compared with traditional learning methods.
S35	Lee and Tucker‐Kellogg [53]	Macromolecular structure–function relationships, focusing on protein structures.	Presents Palantir, an accessible and open‐source mobile application for augmented reality visualization of macromolecules. It aims to enhance the teaching and learning of protein structures in STEM education by making complex molecular visualizations more interactive and engaging.
S36	Loveys and Riggs [54]	Animal & Plant Biochemistry	Describes the successful implementation of the flipped classroom pedagogy in second‐year science laboratory courses at the University of Adelaide, showing significant improvements in student engagement and learning outcomes through online pre‐laboratory activities.
S37	Melo and Morelli [55]	Membrane transport	Stop‐motion animations are an effective strategy for teaching high school students about the transport of substances across cell membranes. This method increased student engagement and comprehension, making abstract biological concepts accessible and engaging through hands‐on learning and digital creativity.
S38	Miles and Soares da Costa [56]	Biochemistry	Investigated the acceptance of clickers in a biochemistry class, finding them beneficial and engaging for both internal and Distance Education students, suggesting their continued use and potential for further integration of new technologies
S39	Mitchell and Means [57]	Protein structure and ligand binding	Elucidates the role of cation−π interactions in biochemistry, providing educational resources and practical exercises to enhance undergraduate biochemistry education.
S40	Monteiro and Araújo [58]	Biochemistry	The effectiveness of the flipped classroom methodology in enhancing the teaching and learning process of biochemistry, particularly with the use of online tools, highlights its benefits in promoting active learning and catering to diverse learning styles.
S41	NPTEL [59]	Online Open Source Courses on Biochemistry I, Biochemistry II, Experimental Biochemistry, and Bio‐inorganic Chemistry.	The National Programme on Technology Enhanced Learning (NPTEL), an online platform funded by the Ministry of Education, Government of India, offers high‐quality, open‐access courses—such as *Experimental Biochemistry* and *Biochemistry I*—featuring video lectures, animations, and comprehensive content in English. These resources make a significant contribution to biochemistry education and are widely used in online learning environments.
S42	Peterson et al. [60]	Biochemistry visualization of three‐dimensional biomolecular structures	Examines the use of augmented reality to teach 3D biomolecular structures, demonstrating increased student interest and improved comprehension through immersive and interactive AR experiences.
S43	Pulukuri and Abrams [7]	Biochemistry bioenergetics	A series of active‐learning videos designed to address misconceptions about energy and free energy in biochemistry by reviewing fundamental chemistry concepts and applying them to cellular reactions, demonstrating significant improvement in student understanding.
S44	Rahmah et al. [61]	Biochemistry Laboratory (*identification of carbohydrates, fats, proteins, and observing enzyme activity*)	Investigates the learning difficulties faced by students during online biochemistry practicums during the COVID‐19 pandemic, revealing significant challenges related to material mastery, practical implementation, internal factors, and external conditions, and emphasizing the need for suitable teaching methods and media.
S45	Romero et al. [62]	Biopharmaceutics and drug design, focusing on the integration of structure‐based design with ADMET (absorption, distribution, metabolism, excretion, and toxicity) principles	Discusses the use of GastroPlus and ADMET Predictor software in a drug design course to teach biopharmaceutics and ADMET principles, highlighting the integration of computational tools to enhance student understanding and practical skills in drug design.
S46	Rowe [63]	Bioinformatics focuses on protein analysis, specifically green fluorescent protein (GFP)	The bioinformatics lab experiment focused on GFP and was aimed at undergraduate biochemistry students. It covers protein identification, multiple sequence alignment, and 3D structure visualization, using accessible bioinformatics tools to enhance understanding of protein structure and function.
S47	Safadel and White [64]	Augmented reality (AR) to enhance the teaching and understanding of macromolecular structures	The use of augmented reality (AR) to enhance molecular biology education has demonstrated that AR significantly improves student satisfaction, usability, and spatial understanding compared with traditional 2D methods.
S48	Shaw [65]	Visualization of protein structures for blind and visually impaired students using tactile graphics	TactViz, a plugin for VMD, is designed to produce tactile graphics of protein structures, enhancing accessibility for blind and visually impaired students. The tool supports tactile visualization on refreshable display devices and provides a means for independent exploration of protein structures, promoting inclusivity in STEM fields.
S49	Silva de Alcantara et al. [21]	Biochemistry of COVID‐19	Development and application of a WebQuest to teach the biochemistry behind COVID‐19 during emergency remote teaching. It highlights the effectiveness of WebQuests in enhancing media and information literacy, as well as engaging students in meaningful learning experiences.
S50	Sung et al. [66]	Biochemistry visualization of potassium channel (KscA)	BiochemAR, an augmented reality app, significantly enhances learning and visualization of macromolecular structures in biochemistry courses by providing an easy‐to‐use, interactive 3D tool.
S51	Terrell & Listenberger [67]	Protein structure and function, focusing on the enzyme cyclooxygenase‐1 (COX‐1).	A multi‐week molecular visualization project, utilizing tools such as UCSF Chimera and AutoDock Vina, enhances undergraduate biochemistry students' understanding of protein structure and function while building their confidence in using computational tools.
S52	Thibaut and Schroeder [68]	Biochemistry Laboratory *(Amino Acid Identification)*	Laboratory techniques and experimental analysis in biochemistry, focusing on case‐based learning (CBL) methods.
S53	Toscanini et al. [69]	Protein structure and function	iMovie Lite, an augmented reality molecular visualization application, significantly enhances undergraduate biochemistry students' understanding of protein structure and function through interactive, hands‐on learning activities.
S54	Uppal and Uppal [70]	Nucleotide metabolism and DNA repair mechanisms	The flipped jigsaw method effectively enhances medical students' understanding of clinical biochemistry by fostering active participation, peer teaching, and self‐directed learning.
S55	Van Dyke and Smith‐Carpenter [71]	Biochemistry Lab (*kinetics experiment, enzyme isolation and characterization, chromatography, absorption, simulation of an acrylamide gel experiment, and outcome*)	Digital laboratory notebook (DLN) using Evernote to enhance student engagement and improve data management in an upper‐division undergraduate biochemistry laboratory.
S56	Vargas‐Oviedo et al. [72]	Biochemistry Lab (*hydrolysis of starch, enzyme α‐amylase concentration, reaction time, pH*)	Enzyme activity, focusing on the catalytic activity of salivary α‐amylase in starch digestion during the pandemic
S57	Volodarets et al. [73]	Biochemical education focusing on the use of e‐learning tools during the COVID‐19 pandemic	E‐learning tools, such as Google Classroom and Moodle, effectively support biochemistry education for medical students in a multicultural environment during the COVID‐19 pandemic, despite some technical challenges.
S58	Wang et al. [74]	General Biochemistry	WeChat Official Account (OA) enhances student‐centered learning and interactive communication in medical biochemistry education, improving student performance and satisfaction.
S59	Wang et al. [75]	Biochemistry of potassium channel (KcsA)	Using BiochemAR, augmented reality‐mediated group activities in a biochemistry classroom reveal fluid and multifaceted patterns of equity and inequity in student participation, influenced by social dynamics and access to technology.
S60	Xu et al. [76]	Biochemistry and molecular techniques used in biological and clinical laboratory contexts.	Combined virtual and actual teaching approaches in medical and developmental biology effectively enhance student learning, engagement, and understanding of complex biochemical techniques through a Virtual Simulation Laboratory.
S61	Yap [77]	Protein biochemistry explicitly focuses on the isoelectric point of amino acids.	A digital module‐based experiential learning framework effectively supports biochemistry education by providing flexible and inclusive access to practical skills and a conceptual understanding of protein biochemistry during the COVID‐19 pandemic.

## 3 Technology in Biochemistry Teaching

Our review observed that technology is typically used in the classroom to project images, as a communication tool through pre‐recorded videos and online meetings, and as a search tool for students [28, 32, 49, 56, 60, 70]. Most articles (89.8%) were developed for higher education, while only 6.78% were designed for high school students and their environments, and 3.39% targeted both the general public and high school students. Results highlight a significant gap in technology use between higher education and high school, with research on technology being more prevalent and frequent in higher education.

**FIGURE 2:**
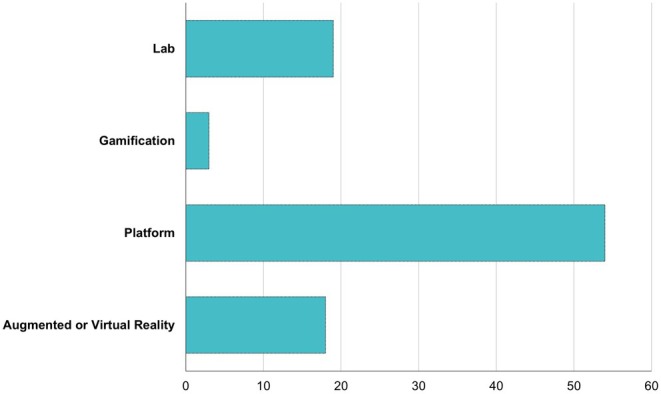
Bar chart illustrating the frequency distribution of technologies identified in the reviewed articles, categorized into online labs and simulators, games and online gamification, educational platforms, and augmented reality.

Access is another crucial aspect to consider. Most works discussing the use of technology in biochemistry teaching (61.9%) come from well‐developed countries such as the USA, among others. When categorizing the tools observed in the articles, we identified the use of smartphones and apps, online labs and simulators, games and online gamification, educational platforms, and augmented reality. Figure 2 shows the frequency of these technologies among the evaluated articles. The graphic does not consider the pandemic because the value showed no strong statistical correlation with Pearson's correlation coefficient between the pandemic and specific digital technologies. However, the platform used was increased and described as a tool. Thus, we understand that studies on the pandemic using Biochemistry and digital technologies should be observed and discussed as a specific topic.

### 3.1 Augmented Reality

As discussed before, abstract concepts to students (Figure 1a) or teaching 3‐D structures and molecular pathways require imaginative thinking, which is often a struggle that overwhelms students, as traditional methods, such as textbooks and static 2D images, fail to convey the dynamic and intricate nature of biomolecules, leading to conceptual gaps. Augmented Reality (AR) and Virtual Reality (VR) are often utilized as digital technologies to enable students to interact with 3D models in real‐time, allowing them to manipulate these models, rotating and examining them from different angles—thereby gaining a more intuitive understanding of spatial relationships and functional sites. Among the articles evaluated in this review, 30% utilize Augmented Reality and Virtual Reality as tools for teaching biochemistry.

AR's media characteristics, namely sensory immersion, navigation, and manipulation, promote positive emotions while learning and create more efficient and better learning outcomes [80]. This hands‐on approach significantly improves spatial reasoning and cognitive skills, making abstract concepts more tangible and comprehensible [81]. By enhancing visualization and interaction with complex molecular structures, AR bridges the gap between theoretical knowledge and practical understanding, making learning more engaging and effective [81]. While these articles are primarily directed at students, they effectively underscore the intrinsic connection between research and learning within the field of Biochemistry. From a research investigative standpoint, such resources contribute to rendering complex biochemical processes more comprehensible and intellectually stimulating. Furthermore, exploring how they nurture critical thinking, encourage inquiry‐driven learning, and integrate theoretical knowledge with practical application—particularly within the realm of scientific communication—adds meaningful analytical depth to the discussion.

Mitchell and Means [57] utilized capture software to visualize online 3D applications, allowing students to explore protein X‐ray structures and discover how cation‐pi interactions stabilize protein structures and protein‐ligand complexes, addressing a topic often neglected by textbooks. Lee and Tucker‐Kellogg [53] utilized Palatir to elucidate macromolecular structure–function relationships, with a focus on protein structures and structural homology. They emphasize the use of an open‐source license, making it publicly available for community testing, improvement, modification, or distribution [53]. Lee and Tucker‐Kellogg [53] also noted that using Augmented Reality was exciting for some students who posted “selfies” with proteins on social media, indicating high engagement in a topic often referred to as overwhelming.

Safadel and White [64] discuss how AR may influence individual attitudes toward computers, such as computer playfulness and anxiety. AR's interactivity enables and challenges users, supports gamified stories, and reveals the unseen, aligning with crucial elements of intrinsic motivation and enhancing learning activities [64]. Toscanini et al. [69] found that student excitement can be leveraged to encourage the exploration of protein structures in their spare time.

Future AR applications should consider options for atomic, space‐filling, or stick renderings and distinguish separate protein subunits by shading them with different colors [53]. Studies by Ibáñez and Delgado‐Kloos [80] explore the cognitive and motivational impacts of AR‐based learning tools. Ibáñez and Delgado‐Kloos [80] found that AR significantly improves students' spatial ability and conceptual understanding.

One downside of this technology is its cost, which can be unrealistic for countries where currency conversion and import taxes significantly increase prices [60]. Additionally, access to devices such as HoloLens AR visors is limited in many countries, with only 16.6% of studies using AR in biochemistry coming from developing countries. It is crucial to consider socio‐economic disparities that impact access to educational resources in developing countries [82]. Solutions such as open‐source software and low‐cost AR applications could help mitigate these issues.

Teaching biochemistry through augmented reality (AR) raises several specific concerns. One major issue is digital equity, as AR requires advanced devices, such as high‐end smartphones or AR glasses, which may not be accessible to all students, particularly those from underserved communities. Sung et al. [66] used AR as a learning strategy, allowing students to reflect on their learning experiences and provide additional information to their original answers. They highlighted the positive use of AR in smartphones, where students can download the app, alleviating the burden of obtaining hardware. For Toscanini et al. [69], biochemistry teachers should utilize portable devices to incorporate meaningful learning activities, especially in resource‐limited settings. However, in third‐world countries, robust technological infrastructure, including high‐speed internet and compatible devices, may be inadequate in many regions, especially developing countries. Students often need smartphones for pedagogical activities that require one, which can create embarrassing situations. Therefore, developing inclusive strategies that do not rely solely on students' devices is essential. Also, investing in technological infrastructure in schools and universities is crucial to ensure that all students can benefit from AR technologies.

Another concern is the preparation and training of teachers. Most of our studies have shown the use of AR by higher education instructors. We ask ourselves if schoolteachers at the high school level and other educators may need to be adequately prepared to integrate AR into their biochemistry classes, leading to an ineffective use of digital technology. Continuous professional development programs focusing on best practices for incorporating AR into the biochemistry curriculum are essential. Furthermore, the cost and sustainability of AR programs pose significant challenges, particularly in institutions with limited budgets. Seeking low‐cost or open‐source AR solutions and forming partnerships with digital technology companies can help mitigate these costs. Should science laboratories not be equipped with and include technological devices?

AR can also create a fragmented learning experience if not integrated with traditional teaching methods. Students may need help to connect what they learn through AR with the rest of the curriculum. Developing an integrated curriculum that combines AR with traditional teaching methods ensures a cohesive and continuous learning experience. This was not a concern observed in our review, but we understand it to be an essential issue. While AR has the potential to enhance student engagement, it can also become a distraction if not well managed. Ensuring AR activities are aligned with learning objectives and require active student participation can maximize the educational benefits of AR.

While AR offers innovative and engaging ways to understand complex biochemical processes, it also presents significant challenges that must be addressed. Ensuring digital equity, improving technological infrastructure, preparing educators, managing costs, and effectively integrating AR into the curriculum are essential for successfully implementing augmented reality in biochemistry education.

## 4 Online Platforms

Platforms are a crucial part of the communication web that envelops everyone, facilitating personal interactions and marketing, e‐commerce, education, cultural creativity, media distribution, entertainment, health applications, and sociopolitical activism [83]. In this context, our review observed that most articles (Figure 2) utilized some form of an online platform.

In biochemistry education, online platforms have become increasingly significant, offering numerous benefits that enhance the teaching and learning experience. We found that the authors referred to using platforms to facilitate critical activities, including interactive learning, resource accessibility, and collaborative work, many of which described platforms as contributing to biochemistry education [66, 72, 84]. For many authors, platforms significantly enhance student engagement by offering interactive content and activities, allowing educators to address questions during lectures and obtain real‐time feedback, like clickers in traditional classrooms. Similarly, platforms like MoleculARweb utilize augmented reality to bring molecular structures to life, enabling students to visualize and interact with complex biochemical processes safely and engagingly [85]. Another noteworthy initiative is the National Program on Technology Enhanced Learning (NPTEL), supported by the Ministry of Education, Government of India. This open‐access platform offers a range of biochemistry‐related courses, including *Biochemistry I*, *Biochemistry*, and *Experimental Biochemistry*, delivered by faculty from institutions such as IIT Kharagpur and IIT Madras. Aimed primarily at undergraduate students in biochemistry, molecular biology, chemistry, and related disciplines, these courses provide structured English‐language content comprising weekly video lectures, theoretical frameworks, experimental demonstrations, and graded assessments.

Online platforms are often used passively, functioning primarily as repositories of curated materials that mirror traditional classroom environments rather than transforming them. This reveals the need for greater pedagogical intentionality in the use of digital technologies. Questions about how students access, engage with, and make sense of content are frequently left unexamined. While NPTEL [59] offers self‐assessment tools, many other platforms lack clear learning parameters and structured feedback. As Feenberg [13] argues, this reflects a dominant logic of instrumental rationality in educational technology, where efficiency and scalability take precedence over dialogue, reflection, and deeper intellectual engagement. Even when designed to address unequal access, evaluations based solely on multiple‐choice questions tend to oversimplify learning and neglect formative dimensions essential to biochemistry education. From a critical perspective, assessments should not only address infrastructural challenges but also support meaningful and contextually relevant learning. Although digital platforms offer innovative ways to explore biochemical content, they often reproduce traditional pedagogical models and perpetuate structural inequities, including language barriers. Their integration into science education must therefore be critically examined to ensure they genuinely contribute to inclusive and transformative learning.

The mass adoption of digital platforms can lead to the homogenization of educational experiences, potentially erasing the diversity of student needs and contexts [86, 87]. Therefore, a nuanced approach to adopting digital technologies, prioritizing equity and inclusion in educational policy and practice, is necessary. Educators using digital platforms should be aware of the potential for these tools to homogenize and diminish diversity [86, 87].

Cerny et al. [88] highlight that companies owning these platforms require users to accept the terms and conditions to use their products. If users disagree, they will be unable to access the platforms. In biochemistry, many higher education professors bring discoveries from their laboratories into the classroom. However, privacy and intellectual property rights for scientific content are often overlooked. This review did not find these discussions prevalent among the authors, yet we consider it relevant to ask: Are platforms ensuring this ownership and privacy?

Haile [43] discusses how topics such as the biochemistry of food can be explored publicly through blogs, thereby enhancing scientific writing and connecting students with their parents and other educators. To connect the topic to a daily experience and make biochemistry a less daunting experience. Pereira‐Dias and Espíndola [12] used food as a basis to discuss diet from different cultural perspectives, critically bringing biochemistry, culture, and diet issues into the discussion for students. In biochemistry education, educators should further emphasize the accessibility of resources and the importance of collaborative work. Additionally, the accuracy and veracity of data found on the internet should be critically evaluated based on scientific parameters such as data reproducibility and biostatistical validation.

Kinsella et al. [51] point out that not all materials available in virtual learning spaces are automatically utilized by students. Our study found that most platforms used were primarily in English, highlighting the risk of homogenizing educational experiences, neglecting individual student needs, and depersonalizing teaching, which can lead to reduced motivation and engagement. To address these issues, developing pedagogical approaches that personalize learning experiences, ensure equitable access to technology and language, maintain high‐quality content, protect data privacy, and foster engagement through interactive elements like quizzes, simulations, and augmented reality is crucial.

Monteiro and Araújo [58] emphasize the importance of guiding students on where to search for reliable information and providing a better framework for learning outside the classroom. Online platforms can enable students to learn content before lectures; a crucial aspect of effective learning.

Nevertheless, these platforms raise concerns, particularly regarding digital equity, as many students require more consistent access to high‐speed internet or adequate devices, resulting in disparities in learning experiences [89]. Ensuring digital equity, improving technological infrastructure, preparing educators, managing costs, and integrating AR effectively into the curriculum are essential to successfully utilizing online platforms in biochemistry education.

## 5 To Lab or Not to Lab: The Use of Digital Technologies and Lab Classes in Biochemistry

Hands‐on laboratory experience is crucial for developing technical skills and competencies in biochemistry. According to Hofstein and Mamlok‐Naaman [90], practical labs enable students to gain proficiency in laboratory techniques, equipment use, and an understanding of experimental procedures. This direct interaction fosters a deep understanding of biochemical concepts and enhances problem‐solving and critical‐thinking skills. Kolb's experiential learning theory (1984) underscores the importance of learning through doing, which is essential for practical labs. Hands‐on experiments enable students to apply theoretical knowledge in a controlled environment, thereby enhancing their understanding of complex biochemical processes. This active engagement makes abstract concepts more tangible and accessible.

Moreover, physical labs promote collaborative learning and teamwork. Working in groups during lab sessions helps students develop communication skills, work effectively with others, and understand different perspectives Hofstein and Mamlok‐Naaman [90]. These social interactions are integral to the learning process and contribute to the development of valuable soft skills.

Lab accessibility is a crucial issue. In this review, 31.6% of the work focused on labs and digital technologies. Key issues emerged from the authors' approach to digital technologies, including integrating digital technologies for hands‐on laboratory experiences, virtual classes during the COVID‐19 pandemic, and virtual lab simulators.

For integrating digital technologies for hands‐on laboratory experience, technology was used to prepare for lab classes, as demonstrated by Loveys and Riggs [54]. Van Dyke and Smith‐Carpenter [91] utilized Evernote to share lab annotations among students, thereby creating a communal environment where instructors interacted with students' annotations while embracing the use of personal devices. This approach addresses the interdisciplinary nature of biochemistry during a hands‐on lab experience. Xu et al. [76] emphasized that combining virtual and hands‐on labs fosters independent, experimental, cooperative, and self‐directed learning, which is fundamental to higher education. When choosing virtual practice tools, Guzmán and Joseph [42] recommended open‐source tools that are compatible with various devices, including tablets, phones, and laptops.

Tatli [92] discussed how virtual labs can simulate hazardous experiments, allowing students to understand risks and safety protocols without exposure to danger, which is particularly beneficial for introductory courses where students learn basic lab safety and procedures. Virtual labs provide accessibility and flexibility, which is crucial when physical lab access is limited, such as during the COVID‐19 pandemic. These tools provide a flexible learning environment that allows students to repeat experiments and explore various scenarios without time or resource constraints.

Our findings indicate that 42% of the articles discussing virtual labs were in the context of the pandemic. The COVID‐19 pandemic necessitated the rapid adoption of virtual labs and online simulations in biochemistry education to compensate for the loss of hands‐on lab access. This shift highlighted the impact of lab accessibility and addressed the inequalities arising from students' physical needs, often excluding them from lab practices. Studies by Cummings [93] and others highlighted the effectiveness of these digital tools in simulating essential biochemistry techniques, such as PCR and gel electrophoresis, providing interactive and engaging learning experiences despite physical lab closures. While virtual labs could not fully replicate the tactile and experiential aspects of traditional labs, they were crucial in maintaining practical skill development and conceptual understanding. They can complement traditional labs by deepening the comprehension of biochemical reactions. Our findings highlighted that the virtual lab, which included instructional videos and interactive simulations, facilitated self‐paced learning and promoted critical thinking and problem‐solving skills [72, 94]. Yap [77] also utilized an online virtual simulator during the pandemic to facilitate experiential learning for students. The laboratory virtual tools were also described as helpful in teaching laboratory calculations, as well as estimations of Protein by Lowry's Method and the Biuret Method, as described in Jeyarajaguru's [48] work.

A summary of the virtual lab simulators found in this review is presented in Table 2. For example, Labster offers virtual lab simulations covering various biochemistry topics, allowing students to conduct experiments in a controlled digital environment [95]. Virtual labs also provide a safe environment for students to conduct experiments that may be too dangerous or costly in a physical lab, benefit high school educators who cannot perform all experiments due to safety issues, and increase access for students with disabilities or phobias. Xu et al. [76] stated that the Virtual Lab is a web‐based educational tool that enables students to fully understand principles and practice by linking them to the theories they have learned. Virtual labs and simulations, such as those provided by Almeida and Valente [96] and Brandt and Novak [97], allow students to perform experiments in a virtual environment, compensating for limited physical lab access. Other examples include ChemCollective, which offers virtual lab simulations that provide scenario‐based problem‐solving exercises for even students with weaker backgrounds to learn [98]. This approach can be helpful to foster a critical connection between theory and practice in Biochemistry. In addition, virtual labs, as presented by Dyke and Smith‐Carpenter [71], simulate real‐world skills, such as students' ability to collect and organize data more efficiently, and demonstrate key pedagogical skills.

**TABLE 2:** An overview of the digital technologies' tools for virtual labs and the biochemistry topics in which the authors specified their use.

Biochemistry topic	Lab simulator	Name of the digital technologies tool
Spectrophotometer Simulation	Biomodel	Spectrophotometer Simulation
Making Stock Solutions from Solid	ChemCollective	Stock Solution Simulator
Solution Stoichiometry	ChemCollective	Solution Stoichiometry Simulator
Creating a Buffer Solution	ChemCollective	Buffer Solution Simulator
Determining the pH of a Buffer Solution	ChemCollective	pH Buffer Simulator
pH Buffer Simulation	University of Oregon	pH Buffer Simulation
Titration Curve of Amino Acids	Vlab Amrita	Amino Acid Titration Simulator
Qualitative Analysis of Carbohydrates	Vlab Amrita, OLabs	Carbohydrate Analysis Simulator
Qualitative Analysis of Amino Acids	Vlab Amrita	Amino Acid Analysis Simulator
Qualitative Analysis of Lipids	OLabs	Lipid Analysis Simulator
Quantitative Estimation of Glucose (Anthrone Method)	Vlab Amrita	Glucose Estimation (Anthrone Method)
Quantitative Estimation of Glucose (DNS Method)	Vlab Amrita	Glucose Estimation (DNS Method)
Quantitative Estimation of Amino Acids (Ninhydrin Method)	Vlab Amrita	Amino Acid Estimation (Ninhydrin Method)
Quantitative Estimation of Protein (Lowry's Method)	Vlab Amrita	Protein Estimation (Lowry's Method)
Determination of Saponification Value of Oils	Vlab Amrita	Saponification Value Simulator
Protein Structure Visualization	JSmol	Protein Workshop
Anaerobic Digestion	EJsS	Anaerobic Digestion Simulator
Various Biochemistry Topics	Phet Colorado	Carbohydrates, among other topics

Despite their advantages, virtual labs are generally seen as supplementary tools rather than complete replacements for physical labs. Brinson [99] noted that while virtual labs can enhance understanding and provide valuable learning experiences, they lack the tactile feedback and real‐world complexity of handling actual materials and instruments. The physical manipulation of lab equipment and real‐time observation of reactions are experiences that virtual simulations cannot fully replicate. Therefore, the tactile and sensory feedback provided by physical lab equipment, as well as experiential learning in a real‐world setting, is irreplaceable. An integrated approach combining virtual and physical labs should be considered to provide comprehensive biochemistry education.

Guzmán and Joseph [42] highlighted the accessibility of EJsS for implementing a class on biochemistry anaerobic digestion, noting that it does not require deep programming knowledge, making it attractive for teachers and lecturers in various disciplines. Their work showed EJsS to be student‐friendly, though students' feedback was not evaluated [42]. Although biochemistry involves many abstract concepts, using 3D models can make molecules come to life. Howell et al. [46] demonstrated that visualizing molecular structures facilitates students' understanding and connection to biochemical functions. Similarly, hand‐held physical models enhance understanding of protein molecules' sites, folding, peptide bonds, and essential concepts. Described by the students as a positive learning methodology [46].

Laboratory drug software is essential for educational applications that incorporate biopharmaceutics and ADMET (absorption, distribution, metabolism, excretion, and toxicity) principles. Romero et al. [62] discussed the use of Gastroplus and ADMET predictors for course design. However, this discussion could be broader by questioning the necessity of using animals for undergraduate courses. The simulation of in silico methods should be discussed when it involves animal lives, potentially presenting a new ethical approach alongside software that brings new outcomes.

There remains a need for scientists trained in the use of this and similar software. Although studies assess students' reception of virtual labs differently, making it unfeasible to determine the best virtual lab software, according to Jeyarajaguru's [48] work, 60% of the students described the virtual resources (Table 2) as excellent. Students should be prepared to know and question the models, works, and data that the software is working on. Rowe [63] raises essential discussions that diminish the salvational use of technology. This result is not surprising, considering that annotating a protein in the JSmol viewer or Protein Workshop requires remembering several specific steps and scripting commands that could be more intuitive, even for a technologically savvy audience. When using lab simulators, several components on the computer running these programs must be set appropriately: pop‐up blockers must be turned off, and Java must be installed and updated [63]. The fact that virtual labs, as presented by Dyke and Smith‐Carpenter [71], simulate fundamental world skills, such as students' ability to collect and organize data more efficiently.

In conclusion, while digital technology tools offer accessibility, safety, and flexibility in biochemistry education, their integration has flaws. Thus, critical thinking is necessary when using virtual labs, because these tools can reduce complex learning to simulations, instrumentalize education, and exacerbate the digital divide. They may alienate students from material reality, promote technological determinism, and diminish collaborative learning dynamics. Thus, it is not a matter of one versus the other. Still, rather than a call to first identify the tools and the author's experience, it is to point out that when choosing an online method, there is a requirement for a pedagogical intention. Additionally, digital technology tools often emphasize standardization over individualized learning. A balanced approach that integrates the strengths of both virtual and physical laboratories, while also addressing their respective limitations and potential inequities, such as limited internet access in Global South countries, is essential for delivering a comprehensive and practical education in biochemistry. In this context, offline laboratory simulators can serve as a valuable resource, as is the case with PhET Colorado (Table 2). Another critical point to consider is the importance of connecting theory to practice as a criterion when using a virtual lab approach in biochemistry [48, 98].

## 6 Gamification With Digital Technologies

Game‐based learning tools have significantly enhanced engagement and understanding in the field of Biochemistry Education. Silva et al. [100] highlighted incorporating educational games into the curriculum to make learning more interactive, emphasizing the need for tools that transform passive learning into an active, student‐centered process; this is especially relevant given that one of the significant issues in biochemistry education is the subject's voluminous and sometimes tedious nature [7]. However, only 5% of the articles reviewed used digital technologies and gamification.

Gamification can be an effective tool to maintain student interest and engagement, provided it is carefully integrated into the curriculum with a focus on enhancing content mastery. Gamification is helpful due to the voluminous aspects of Biochemistry Teaching. For example, Silva de Alcantara et al. [21] developed “BioDomínio,” a gamified learning tool resembling Monopoly, which helped students comprehend biochemical pathways in a fun and interactive way. Future studies should rigorously evaluate these tools over extended periods to confirm their efficacy in improving academic performance and conceptual understanding.

While adopting these gamified tools was primarily driven by the need to engage students, it is essential to consider how such technologies can transform pedagogical practices. As Espíndola and Grané [101] emphasized, gamification should be critically assessed for its potential to promote or hinder educational equity. The socio‐political implications of gamification are significant. Many online game tools are commercial products, limiting access for students from lower socio‐economic backgrounds, and are often available only in dominant languages, excluding non‐native speakers. Addressing these issues requires a commitment to developing open‐access, multilingual educational resources.

Ethical considerations are paramount when integrating gamification into the education of biochemistry. The uncritical use of gamified tools may necessitate a greater focus on significant issues, such as data privacy and the digital divide [102]. Zuboff [82] highlights concerns about surveillance and data exploitation in educational settings, particularly relevant for gamification platforms that collect and analyze student data. To mitigate these risks, it is crucial to establish stringent data privacy policies and ethical guidelines, ensuring transparency regarding data usage and providing students with control over their personal information.

To ensure that gamification serves as an inclusive and equitable educational tool, it is necessary to design these platforms with accessibility in mind, ensuring all students can participate fully in gamified learning activities. The Inclusive BioChem software exemplifies this approach by incorporating text‐to‐speech and audio descriptions, interactive simulations with adjustable difficulty levels, keyboard and voice controls, high‐contrast and color‐blind modes, and customizable learning paths. More inclusive software should be developed, incorporating universal design principles and made accessible in various languages to ensure that all students, regardless of their disabilities or linguistic backgrounds, can fully engage with and benefit from gamified learning tools. Addressing broader systemic barriers, such as the digital divide, is essential for true inclusivity.

In conclusion, the integration of gamification in biochemistry education holds significant potential for enhancing student engagement and learning outcomes. However, critically examining its ethical and socio‐political implications is essential to ensure these tools contribute to inclusive and equitable educational experiences. The urgent deployment of digital technologies during the pandemic highlighted their indispensable role, but also overlooked issues of digital equity and access disparities. Feenberg [103] and Selwyn [14] remind us that educational technologies must be evaluated for their practical benefits and capacity to reinforce or mitigate existing inequalities. In the Brazilian context, while educational games and interactive simulations have improved engagement and learning efficiency [12], they frequently lack a critical perspective on equity and inclusion. Therefore, moving forward, it is crucial to strike a balance between the practical advantages of digital technologies and a thorough examination of their socio‐political impacts. Ensuring that gamification in biochemistry education promotes equity and inclusivity for all students requires continuous evaluation and adaptation of these tools to address emerging ethical, cultural, and accessibility challenges. Souza Junior et al. [49] describe structural gamification, where the entire didactic sequence of SAI incorporated a game‐scoring mechanism to encourage student engagement and found that it promoted collaborative learning.

## 7 Biochemistry and Digital Technologies Used During the COVID‐19 Pandemic

The COVID‐19 pandemic has accelerated the integration of digital technologies into biochemistry education, reshaping how educators and students engage with the subject. The rapid shift to online learning platforms necessitated by the pandemic has brought both opportunities and challenges to the forefront. However, this transition also necessitates a critical examination of the broader socio‐political implications of these tools.

**FIGURE 3:**
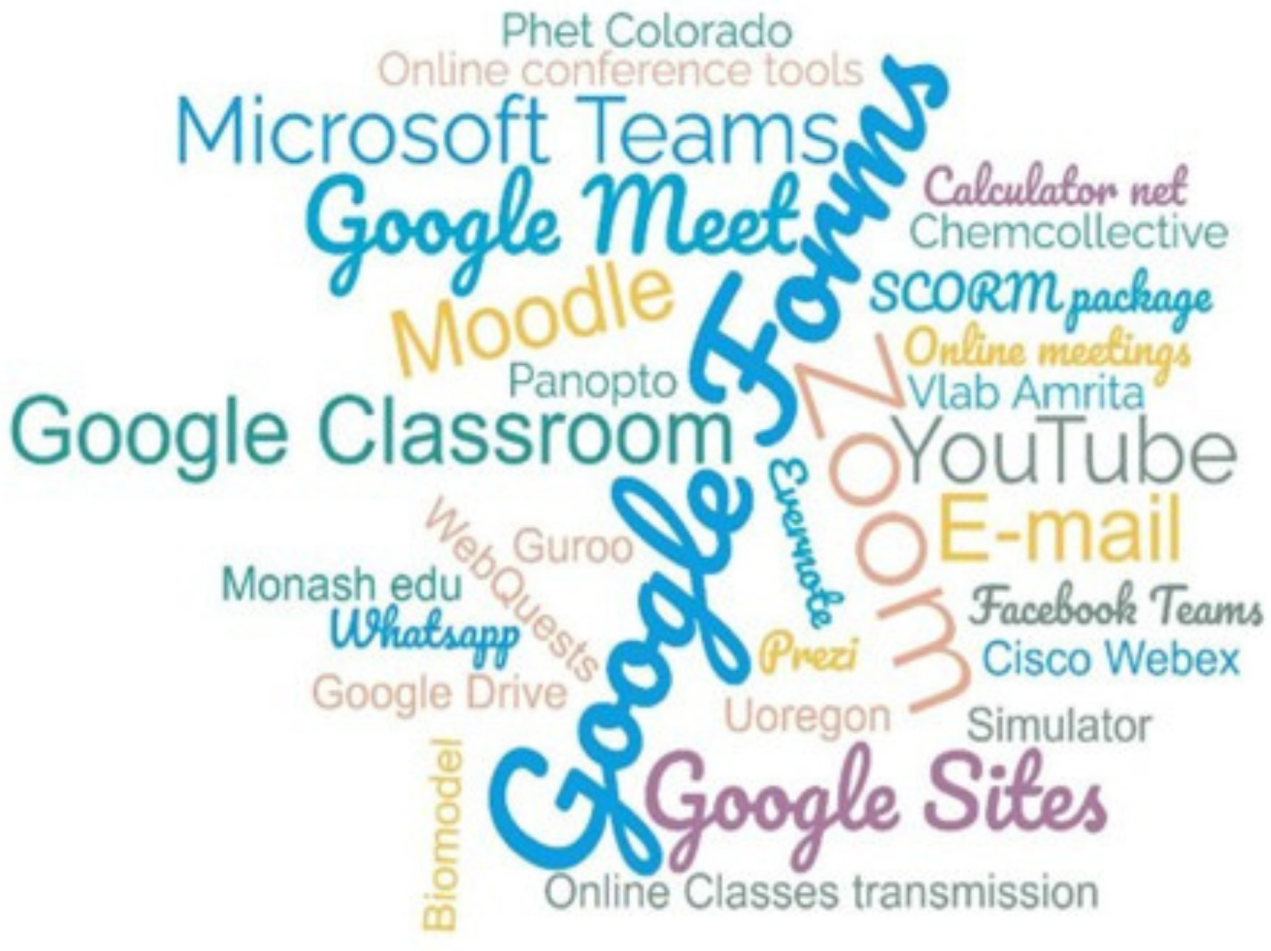
Word cloud illustrating the digital platforms and tools employed in studies on biochemistry teaching during the COVID‐19 pandemic. The prominence of digital technology platforms, such as Google Suite, Microsoft and Zoom, is evident in the research conducted during this period.

Among the works conducted during the pandemic, 40% of the studies have utilized or considered smartphone access as a tool. Figure 3, a word cloud, shows all the tools used. The impact of digital technology tools on biochemistry education during the pandemic is evident from various studies; most studies (86.67%) utilized digital technology platforms. Among the platforms used, Google Suite tools (forms, meet) or other tools [21, 36, 41, 48, 61, 72, 77, 104] were the most used (86%), followed by Microsoft [21, 36] and Zoom [41, 73, 77]. YouTube platforms were also present in many works [61, 73]. This data suggests using digital technology tools for online communication during isolation.

While these tools were necessary for maintaining educational continuity, their use was primarily instrumental, aimed at replicating traditional classroom settings online without critically addressing issues such as digital equity and access disparities [89]. Relying on platforms like Google Suite, Microsoft, and Zoom raises concerns about the potential for commercial interests to influence education. The implications of outsourcing educational infrastructure to private companies and the impact on data privacy and autonomy must be critically examined.

Figure 2 illustrates the diverse range of digital platforms and tools used to support biochemistry education during this period, including virtual labs, interactive simulations, online assessments, and collaborative tools. Volodarets et al. [73] questioned the variability in the effectiveness of tools across different language groups and how to address a multicultural environment even during the pandemic. Language is a significant issue, as many online tools for biochemistry are available only in English. The use of smartphones versus computers was an accessibility observation in the work of Hoog et al. [45]. Yap [77] noted that digital, experiential learning modules offer students a flexible, inclusive, equitable, and objective‐oriented learning environment during the COVID‐19 pandemic.

Thibaut and Schroeder [68] utilized digital technologies as a transmission tool to adapt biochemistry labs to an online format, employing case‐based learning, which effectively mimics essential laboratory qualities and promotes student engagement and collaboration during the COVID‐19 pandemic. Despite these advantages, the rapid deployment of digital technology tools also underscored significant challenges, particularly regarding equity and accessibility. Decuypere et al. [86] and Pretto et al. [87] emphasize the critical need to examine digital education platforms through a lens that considers their socio‐political implications. The findings from Figure 3 reveal that while platforms like Moodle, Zoom, and Google Classroom facilitated the continuation of education, they also highlighted disparities in access to technology and the internet.

Several obstacles have been identified in the literature that hindered biochemistry teaching during the pandemic. Pereira‐Dias and Espíndola [12]; Vasiliadou [105] pointed out that the sudden shift to virtual labs created a steep learning curve for students and instructors unfamiliar with these tools. Additionally, Ma and Nickerson [106] noted that while virtual labs can simulate experiments, they cannot fully replicate the hands‐on experience of physical labs, which is crucial for developing practical skills. Surahman and Wang [107] noted that students from underprivileged backgrounds faced difficulties accessing reliable internet and necessary technological devices, exacerbating educational inequalities. Additionally, Karim and Alam [108] reported new pressures like students and teachers struggling with the abrupt transition to online platforms, citing issues such as inadequate digital literacy and the lack of technical support.

Drawing on the critical perspectives of Espíndola et al. [109], Espíndola and Grané [101], and Feenberg [110], it becomes evident that the creation and integration of digital educational technologies should be viewed as a process of struggle and resistance against dominant educational paradigms. This perspective is crucial when considering the rapid platformization of education during the pandemic. Educators can resist the uncritical adoption of digital tools that may reinforce existing inequalities by promoting critical approaches to educational technology. Espíndola and Grané [101] argue that it is imperative to adopt critical approaches that interrogate the broader socio‐political impacts of these technologies, including issues of digital equity, accessibility, and the potential for reinforcing socio‐economic disparities.

Kamber [50] used an online oral examination to avoid challenges during virtual distance learning. Answer‐sharing websites and online paid “tutoring” services such as Chegg, CourseHero, and JustDoMyHomework allow students to submit questions and ask for help in real time. Online exam proctoring services can minimize cheating, but students are only partially prevented from avoiding monitored browsers, which demands intensive help from assistant professors. Kinsella et al. [51] utilized multiple‐choice questionnaires during screen casting to assess their adequacy for students and the necessity of feedback. Rahmah et al. [61] argued that learning media facilitated learning and teaching activities during the COVID‐19 pandemic by providing various communication features and information sharing. Although students were already accustomed to using the internet for social networking or searching for informal students, they felt that specific students were causing learning difficulties.

Vargas‐Oviedo et al. [72] developed a laboratory practice using human salivary α‐amylase to study starch digestion, employing home materials such as flour (cornstarch), physiological serum solution (saline), lemon juice, sodium bicarbonate, and povidone‐iodine solution (Isodine) to conduct online synchronous meetings and laboratory practices during the pandemic. Kubbara et al. [52] demonstrated the necessity of online case‐based learning for biochemistry teaching. However, like other authors in this study, tools of digital technologies were utilized for streaming purposes, and the technology was not examined as a subject of research or an issue to be discussed.

In conclusion, the COVID‐19 pandemic has undeniably accelerated the integration of digital technology tools in biochemistry education, highlighting both the potential benefits and inherent challenges. While these tools have facilitated continuity of learning and enhanced educational engagement, they have also exposed significant issues related to equity and accessibility. Future efforts must focus on developing inclusive, equitable digital learning environments that address the socio‐political dimensions of educational technology, ensuring that all students can benefit from these advancements. The learning media used are sourced from YouTube videos, which sometimes use English as the language of instruction, making it difficult for students to understand the purpose and objectives of the practicum video Rahmah et al. [61]. Volodarets et al. [73] observed difficulties, including submitting handwritten replies and technical issues, when using Google Classroom on laptops and mobile phones. Silva et al. [104] used a WebQuest approach to teach the biochemistry behind COVID‐19 during emergency remote teaching.

## 8 Call to Reflect on the Use of Technology in Biochemistry Education: Beyond a Mere Instrument

**FIGURE 4:**
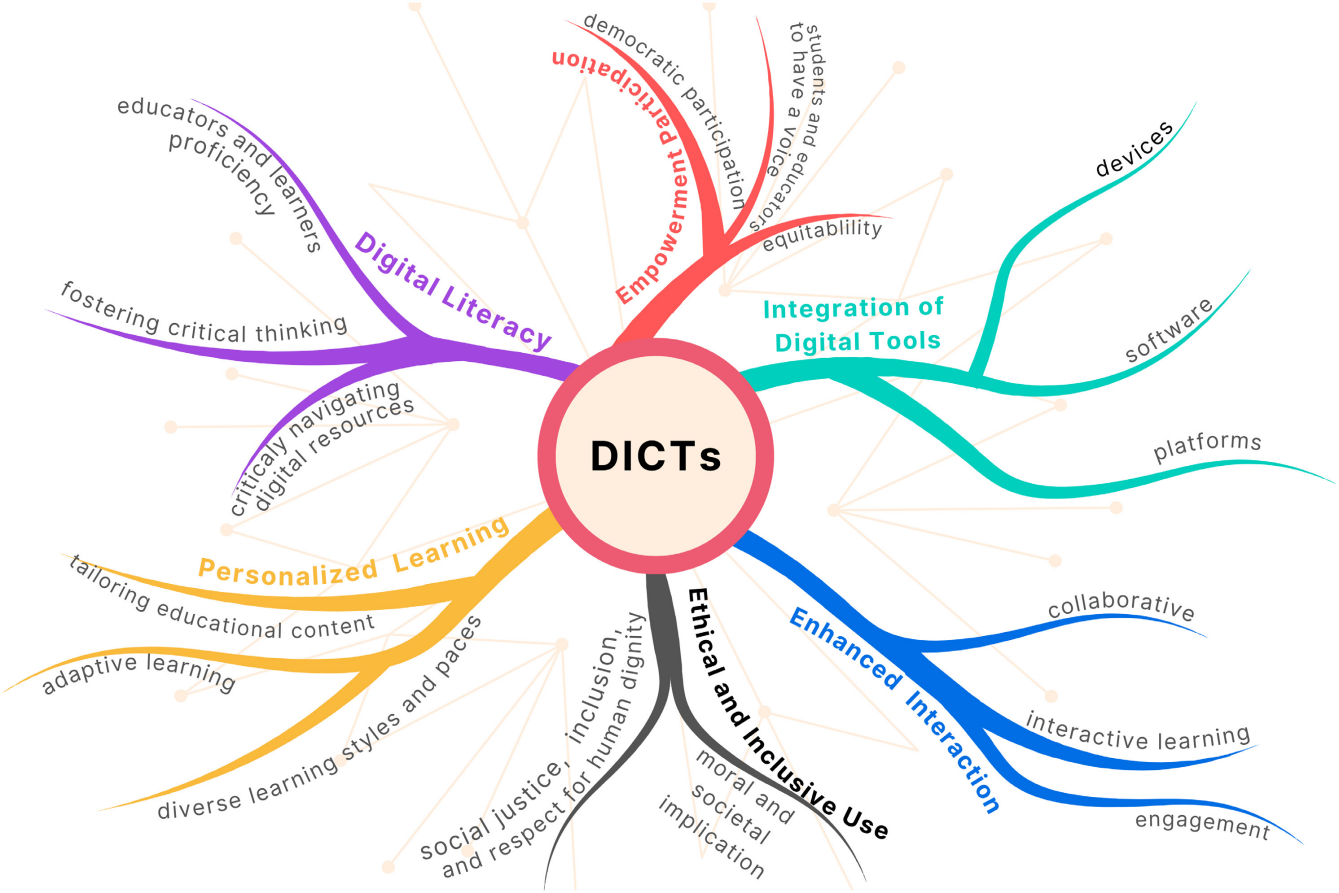
Conceptual map illustrating the multiple dimensions and implications of Digital Information and Communication Technologies (TDICs) in education. The diagram emphasizes that digital technologies should be understood not merely as “tools”, but as complex sociotechnical systems encompassing empowerment and participation, enhanced interaction, digital integration, personalized learning, ethical and inclusive use, and digital literacy; each unfolding into its own interconnected dimensions.

We urge the educational community to explore the use of technology beyond mere instrumental applications in classrooms, believing that Biochemistry Education can significantly benefit from this approach. Integrating Digital Information and Communication Technologies in biochemistry teaching, especially during the COVID‐19 pandemic, has presented opportunities and challenges. While technologies like virtual labs and augmented reality have enhanced accessibility and interactivity, their instrumental use often overlooks broader implications and transformative potential. The integration of Digital technologies in Education should be integrated into learning, promoting collaboration, ensuring digital literacy, and supporting social justice and dignity in a dynamic, rapid, and engaging process, with numerous challenges, as illustrated in Figure 4. According to Wang et al. [75], the introduction of AR in Biochemistry and other technologies is imperative to continue expanding our conception and evaluation of equity beyond access to physical technology itself and performance‐ or knowledge‐based outcomes, considering how the utilization of that technology in the classroom impacts access to opportunities to participate in learning.

For Biochemistry Education, several authors have used technology to address some of the problems found in Figure 1a. Higgins et al. [44] considered the crucial question: Was the tool effective as a learning tool when used on a platform? Such a question should always be considered. These authors observed overall positivity, but a proportion of students found PeerWise ineffective as a learning tool, with one student describing it as “at best an annoyance, at worst a distraction” [44]. Evaluating the quality of other students' questions and receiving feedback are essential aspects of a study. Before using any tool, questions posed by Terrell and Listenberger [67] can be considered necessary. Knowledge encompasses both the awareness that an online database or computational tool exists and the understanding of its specific function. We used our survey to assess both student awareness and understanding separately. We define awareness as familiarity or having previously been exposed to a tool or database. This questioning is essential, as shown by Terrell and Listenberger [67], who found that student responses regarding the Protein Data Bank revealed misconceptions in their understanding of this program tool.

Lawson and Martella [71] raise essential considerations when using Immersive Virtual Reality, expressing concern about cognitive load. The Cognitive load of digital technologies should be considered by educators, especially with many students having ADHD or autism spectrum disorder. Is technology considering all students and creating opportunities, or is virtual reality emphasizing exclusion due to cognitive load or economic gaps? This critical question should be considered before any implementation. Rahmah et al. [61] also note that the vast amount of material was unhelpful and tedious during the COVID‐19 experience.

Melo and Morelli [55] acknowledge the field of digital technologies and use stop‐motion as a strategy for high school students to create and debate their perceptions of biochemistry membrane transport. The aim was to make the student the protagonist of the video construction process. They also acknowledge technology as a part of the BNCC, promoting debates around scientific and/or technological topics of socio‐cultural relevance. Despite this, the technology discussion was more focused on the difficulty of creating and producing the videos than on other aspects, such as platform choice and authorship. For Pulukuri and Abrams [7], videos should not be used in isolation but instead accompanied by questions to make interactions between students and teachers more dynamic while critically analyzing the knowledge and content presented in the videos. This might be an important topic: Is everything posted online accurate? What databases generate this information, and are they reliable sources? Scientific facts should be reproducible and reliable, derived from a statistically relevant population, and acknowledge the field scientists and their work that supports the fundamentals. While students can benefit from these online sources, they should first and foremost know how, when, and what aspects make a good source. Questioning videos is essential to critically engaging with widely available online information.

The work by Shaw and Hadden [65] highlights an important aspect of technology: independence, particularly for young scientists who are blind and seeking ways to participate in STEM fields. Along with 3‐dimensional printed models, variable‐height tactile graphics produced using TactViz enable individuals who are blind or visually impaired to conceptualize and analyze protein structures through the sense of touch [65]. Alberto Cupani critiques the instrumental use of technology, emphasizing that viewing it solely as a tool to achieve predefined objectives often ignores its broader implications [111]. A mechanistic use of technology contrasts with Andrew Feenberg's perspective, which sees technology as fundamentally shaping educational practices and social relations [13]. For instance, Gardner et al. [112] present remote labs, such as Labster, as a means of maintaining educational continuity. These digital technologies simulate laboratory experiences, making them accessible to students who may not have access to physical labs. However, these digital technologies are often used to replicate traditional lab experiences rather than reimagining how students engage with biochemical concepts and encouraging them to critically question underlying scientific principles.

Incorporating a critical perspective on the use of digital technologies in biochemistry education ensures that these tools enhance rather than replace foundational skills, support essential hands‐on laboratory practices, and help prevent the reinforcement of social inequalities and hegemonic structures [113]. To promote the integration of critical thinking, educators can implement the following strategies:

1. Educators should present scientific concepts within broader societal and ethical contexts. For example, when teaching signal transduction pathways, instructors can discuss their implications in medical research and drug development, including the ethical considerations that are important when using technology [114]. Instead of merely following predefined protocols, students should be encouraged to design their experiments, formulate hypotheses, and explore multiple outcomes. Virtual lab platforms, such as Labster, can be used to simulate various scenarios and experimental conditions, allowing students to explore “what if” questions and their implications.
2. Biochemistry intersects with fields like genetics, environmental science, and bioethics. Educators should integrate these disciplines into the curriculum, encouraging students to understand the multifaceted nature of biochemical problems. For instance, synthetic biology topics can be linked to discussions on climate change, food security, and ethical issues, such as the use of animals in research and the creation of in vitro meat [85].
3. Collaborative learning tools can foster teamwork and peer learning. Students can work together on projects that require them to research, discuss, and present complex topics, such as metabolic pathways and their role in health and disease management [115]. Online forums and platforms can simulate real‐world research environments where students must collaborate and critique each other's work.
4. Educators and policymakers must adopt a more critical and inclusive approach to move beyond the instrumental use of technology. Encouraging educators to reflect on the broader implications of technology use in education aligns with Feenberg's and Selwyn's critical approaches to education. Integrating technology to align with the educational project of forming critically thinking individuals who can navigate and challenge existing power structures is essential. Thus, both initial training and continuing professional development of educators within the digital domain are necessary to achieve the meaningful integration of digital technologies in education [109].

During the COVID‐19 pandemic, Pereira‐Dias and Espíndola [12] critically examined the challenges posed by reliance on digital platforms, noting issues such as accessibility and the potential for reduced hands‐on experience. They debated fundamental concepts, including carbohydrates and proteins, which allowed for a deeper understanding of diet not only locally but also globally. This enabled students to comprehend food diets worldwide, question basic paradigms, and engage with movies, websites, and games, promoting the meaningful learning of relevant Biochemistry knowledge.

Similarly, Silva et al. [104] used the COVID‐19 pandemic as a topic to reflect on the virus, creating awareness and interest in biochemistry simultaneously. Using the webcast approach, specifically a WebQuest, they expressed positive perceptions about digital technologies for emergency remote teaching, particularly regarding the sufficiency of materials and resources available on the platform. They also discussed different understandings of digital literacy (technological) and the concept of informational and media literacy.

## 9 Final Considerations and Emerging Challenges for the Use of Digital Technology in Biochemistry Education

This review examined how various authors addressed the challenges of teaching biochemistry using digital technologies. While many proposed innovative strategies, some also highlighted the limitations of these technologies, emphasizing the discipline's complex pedagogical demands, the irreplaceable role of laboratory instruction, and the impact on student learning. As more technology has been adopted in biochemistry classes, we observed that few authors, however, approached the use of digital technology through a critical lens. Future studies would benefit by questioning: Do your digital technologies help students understand how biochemical knowledge is constructed, or do they simply support fact memorization? Who controls how technology is used in your classroom: you, your students, or the institution? These reflections are essential, not to address technological shortcomings per se, but to examine the educational values and practices that these tools promote.

Digital technologies will become a more relevant issue for Biochemistry Educational Studies, as the rapid integration of artificial intelligence (AI) into biochemistry classes reshapes not only the delivery of content but also the creation, valuation, and understanding of scientific knowledge. Biochemistry's inherent complexity, its reliance on abstract reasoning, experimental inquiry, and conceptual synthesis, demands more than a technocratic substitution of traditional methods. Instead, it calls for intentional pedagogy that evaluates how AI mediates understanding, cultivates student autonomy, and supports collaborative learning. Digital technologies and AI hold promises for enabling personalized and cooperative education, provided they are embedded within socially and contextually responsive practices. As Espíndola and Grané [101] argue, their true potential lies not in novelty but in their ability to foster collective inquiry and dialogical engagement. Tools such as Socratic by Google, ChemDraw, Molecular Workbench, and Labster offer adaptive feedback and dynamic environments that can democratize access to high‐quality learning resources [96]. In this evolving landscape, students should be empowered to respond creatively to their learning challenges, treating digital technologies not merely as functional aids but as tools for conceptual problem‐solving and epistemic agency.

The deployment of digital technologies in biochemistry education, most notably simulation platforms, has become emblematic of contemporary pedagogical innovation. These tools aim to replicate experimental conditions and facilitate the translation of theoretical content into practical understanding [85]. Yet this techno‐pedagogical shift is far from neutral, as it could mask structural inequities, reinforce access asymmetries, and, in many contexts, substitute the appearance of modernization for substantive educational reform.

In regions with inadequate digital infrastructure, the rhetoric of digital transformation collapses under the weight of logistical realities. Online classes are often reduced to collective broadcasts in resource‐limited classrooms, a compensatory gesture that does little to rectify underlying disparities. More critically, even the most advanced digital platforms remain incapable of reproducing the tacit, embodied learning intrinsic to laboratory practice. The epistemic and procedural depth acquired through hands‐on experimentation cannot be digitized without loss. To conflate simulation with experience is to dilute the core of scientific training. Addressing these structural deficiencies requires more than technological optimism; it demands deliberate, redistributive interventions. Shared funding mechanisms should support localized, community‐rooted educational infrastructures that integrate digital tools into pedagogies attuned to local material conditions. Faculty development, too, must be reimagined, not as technical upskilling, but as the cultivation of reflective practitioners equipped to examine the epistemological and ethical implications of digital instruction critically.

This imperative is particularly urgent in the Global South and other historically marginalized regions, where legacies of systemic underinvestment continue to exacerbate educational inequalities. The introduction of digital technologies into such landscapes is not merely a matter of access, but also one of epistemic justice. It raises fundamental questions about who defines what counts as valid knowledge, whose pedagogical models are prioritized, and what forms of authority are legitimized in the classroom. In this light, biochemistry education must resist the appeal of technological determinism. The question is not merely which digital technologies should be adopted, but what assumptions those digital technologies carry about cognition, expertise, and the politics of learning. Any genuine advancement in scientific education must address these questions directly, lest innovation become yet another vector of exclusion.

These assumptions matter because digital technology helps define what is recognized as valid scientific practice and who is positioned to participate in it. The growing use of generative AI (GAI), which automates tasks like analysis, prediction, and feedback, marks a shift away from student‐centered cognitive engagement. This trend raises critical epistemological questions: What types of reasoning are being prioritized or displaced? How does such digital technology shape access to scientific literacy and participation? As Zuboff [82] and Selwyn [116] caution, digital systems often reflect broader logics of surveillance, commodification, and dehumanization. In a discipline like biochemistry, where experimentation, iteration, and embodied reasoning are central, these logics risk undermining educational quality and eroding the formative dimensions of scientific inquiry.

A critical pedagogical response requires more than technical adaptation. It demands that educators and institutions examine the normative frameworks that digital technologies reinforce. As Espíndola and Grané [101] emphasize, educational technologies must contribute to inclusive, socially responsive environments. Yet if adopted uncritically, they can displace essential aspects of scientific formation, such as hands‐on manipulation, critical dialogue, and collaborative learning, especially for students from structurally marginalized backgrounds. Meeting these challenges entails a shift in educational paradigms: from instrumental approaches to digital technologies toward a vision grounded in ethical responsibility, curricular integrity, and epistemic justice. As argued by Pereira‐Dias and Espíndola [12] and Espíndola and Giannella [117], this shift involves questioning which knowledges are legitimized, whose experiences are valued, and how inclusion is operationalized through technological mediation. As Ortega y Gasset [118] warned, when technology eclipses the human dimension, education risks becoming alienating. For biochemistry educators, the challenge lies not merely in adopting new digital technology but in articulating a pedagogical vision that aligns technological integration with equity, conceptual rigor, and meaningful scientific engagement. At its core, the question extends beyond how we teach biochemistry to encompass how we envision the future of science education, fostering a more just, critically informed, and human‐centered society in an increasingly technologically advanced world.

## Funding

This research was supported by a PhD scholarship from FAPESC (Fundação de Amparo à Pesquisa e Inovação do Estado de Santa Catarina).

## Ethics Statement

No ethical approval was required, as this is a theoretical study that did not involve human participants or animals.

## Conflicts of Interest

The authors declare no conflicts of interest.

## Data Availability

Data sharing not applicable to this article as no datasets were generated or analyzed during the current study.
